# Reply to “Important Methodological and Statistical Concerns Regarding the Association of Factor V Leiden and MTHFR Polymorphisms With COVID‐19 Outcomes”

**DOI:** 10.1002/iid3.70556

**Published:** 2026-10-09

**Authors:** Hilal Çıralıoğlu, Yasemin Adalı, Mert Özen, Alten Oskay, Atakan Yılmaz, Murat Seyit, Aylin Köseler, İbrahim Türkçüer

**Affiliations:** ^1^ Department of Emergency Medicine Pamukkale University Faculty of Medicine Denizli Türkiye; ^2^ Centre for Public Health, School of Medicine, Dentistry and Biomedical Sciences Queen's University Belfast Belfast UK; ^3^ Division of Epidemiology, Department of Public Health, Faculty of Medicine Pamukkale University Denizli Türkiye; ^4^ Department of Biophysics Pamukkale University Faculty of Medicine Denizli Türkiye

Dear Editor,

We thank Dr. Panda for the careful reading of our article and for raising important methodological and statistical points regarding Hardy–Weinberg equilibrium (HWE) assessment and the reporting of genetic association measures [1]. We agree that these analyses provide useful additional context for interpretation of our findings. We therefore re‐examined the original individual‐level data sets comprising 150 patients with COVID‐19 and 300 controls and provide the results and clarifications below.

**Response to Comment 1: Hardy–Weinberg equilibrium**

We agree that HWE assessment should have been explicitly reported in the original article. In the control group, the FVL G1691A genotype distribution was 261 GG, 39 GA, and 0 AA. The corresponding Pearson HWE test yielded *χ* ^2^ = 1.45 (*p* = 0.229), and the exact HWE test was also non‐significant (*p* = 0.623), indicating no statistically significant departure from HWE. For MTHFR A1298C, the control‐group distribution was 184 AA, 116 AC, and 0 CC. The Pearson HWE test yielded *χ* ^2^ = 17.23 (*p* = 3.31 × 10^−5^), and the exact HWE test likewise confirmed significant deviation from HWE (*p* = 1.26 × 10^−6^). We acknowledge that this finding is an important limitation of the MTHFR case–control comparison and that it should have been explicitly reported and discussed in the original article.

A deviation from HWE in controls can arise from several sources, including population structure, selection effects, sampling characteristics, or genotyping‐related factors. Accordingly, the association between MTHFR A1298C and case–control status should be interpreted cautiously and should not be taken as evidence of a protective effect without independent replication. As described in the original Methods, the relevant polymorphic loci were evaluated following PCR amplification and high‐resolution agarose gel analysis, and the genotype calls were subsequently confirmed by DNA sequence analysis. The published report did not separately specify the use of duplicate samples or negative controls; therefore, we do not retrospectively claim quality‐control procedures that were not explicitly documented. The available data do not allow us to determine the specific reason for the observed HWE deviation.

In light of this limitation, the association observed between MTHFR A1298C and COVID‐19 case–control status should be interpreted with caution and cannot be considered robust evidence of a protective association without confirmation in an independent population.

At the same time, the scope of this limitation should be clearly distinguished from the within‐patient analyses. Importantly, the HWE deviation was identified in the healthy control group and therefore primarily affects the interpretation of the between‐group MTHFR association. It does not, by itself, invalidate analyses performed within the COVID‐19 cohort examining relationships between genotype and clinical outcomes.

**Response to Comment 2: Allele frequencies, odds ratios, confidence intervals, and genetic models**

We agree that odds ratios (ORs) with 95% confidence intervals (CIs), together with allele frequencies, are more informative than p values alone for a case–control genetic association analysis. Reanalysis of the original data confirmed the calculations highlighted in the Letter.

For FVL G1691A, the GA genotype was observed in 34 of 150 patients with COVID‐19 (22.7%) and 39 of 300 controls (13.0%). Relative to GG, GA was associated with higher odds of belonging to the COVID‐19 group (OR = 1.96, 95% CI 1.18–3.26; Fisher's exact *p* = 0.010). The A‐allele frequency was 11.3% in patients and 6.5% in controls, with an allele‐based OR of 1.84 (95% CI 1.13–2.98; *p* = 0.014).

For MTHFR A1298C, the AC genotype was observed in 35 of 150 patients (23.3%) and 116 of 300 controls (38.7%). Relative to AA, AC was associated with lower odds of belonging to the COVID‐19 group (OR = 0.48, 95% CI 0.31–0.75; Fisher's exact *p* = 0.0014). The C‐allele frequency was 11.7% in patients and 19.3% in controls, corresponding to an allele‐based OR of 0.55 (95% CI 0.37–0.83; *p* = 0.0034). However, because the MTHFR genotype distribution in controls departed significantly from HWE, both the genotype‐ and allele‐based MTHFR estimates should be interpreted cautiously.

These effect estimates should, however, be interpreted in conjunction with the HWE findings described above. The significant HWE deviation observed for MTHFR A1298C in the control group limits the strength of the case–control inference. We therefore consider the MTHFR case–control findings exploratory and believe that they require confirmation in an independently recruited population before a protective association can be inferred.

No FVL AA or MTHFR CC homozygotes were observed in either group. Consequently, homozygote‐specific or recessive genetic models could not be meaningfully evaluated in this data set (Table 1).

**Table 1:** Reanalysis of FVL G1691A and MTHFR A1298C polymorphisms in patients with COVID‐19 and controls using the original data set reported by Çıralıoğlu et al. [2]

Polymorphism	Comparison	COVID‐19	Controls	OR (95% CI)	*p* value	HWE in controls
FVL G1691A	GG	116/150 (77.3%)	261/300 (87.0%)	Reference	—	*χ* ^2^ = 1.45; *p* = 0.229; exact *p* = 0.623
	GA	34/150 (22.7%)	39/300 (13.0%)	1.96 (1.18–3.26)	0.010	
	A allele	34/300 (11.3%)	39/600 (6.5%)	1.84 (1.13–2.98)	0.014	
MTHFR A1298C	AA	115/150 (76.7%)	184/300 (61.3%)	Reference	—	*χ* ^2^ = 17.23; *p* = 3.31 × 10^−5^; exact *p* = 1.26 × 10^−6^
	AC	35/150 (23.3%)	116/300 (38.7%)	0.48 (0.31–0.75)	0.0014	
	C allele	35/300 (11.7%)	116/600 (19.3%)	0.55 (0.37–0.83)	0.0034	

*Note:* Genotype denominators are numbers of participants; allele denominators are total numbers of alleles (two alleles per participant). ORs compare the variant heterozygous genotype with the reference homozygous genotype, or the variant allele with the reference allele. ORs and 95% CIs were calculated from 2 × 2 contingency tables using the log‐odds (Wald) method; *p* values were obtained using two‐sided Fisher's exact tests. HWE was assessed in controls using both the Pearson *χ* ^2^ test with 1 degree of freedom and an exact HWE test.

**Overall interpretation**

We appreciate Dr. Panda's comments regarding HWE assessment and the reporting of effect estimates. The additional analyses support the direction of the originally reported FVL G1691A case–control association. For MTHFR A1298C, although the numerical association remains evident in the reanalysis, the significant deviation from HWE in the control group substantially limits its interpretation. We therefore believe that the MTHFR finding should be regarded as preliminary and interpreted cautiously until replicated in an independent population.

These comments have also highlighted areas that should be addressed more explicitly in future genetic association studies. Future studies should include prospectively defined genotyping quality‐control procedures, explicit HWE assessment, complete reporting of genotype and allele frequencies, and effect estimates with confidence intervals, preferably in larger and independently recruited populations.

We thank Dr. Panda for raising these methodological and statistical points and for providing us with the opportunity to clarify the interpretation of our findings.

## Author Contributions

**Hilal Çıralıoğlu:** conceptualization, investigation, funding acquisition, writing – original draft, writing – review and editing, visualization, validation. **Mert Özen:** software, resources, data curation, formal analysis, project administration. **Yasemin Adalı:** writing – original draft, software, writing – review and editing, supervision, project administration, conceptualization, investigation, visualization. **Mert Özen:** conceptualization, investigation, funding acquisition, writing – review and editing, visualization, validation. **Mert Özen:** resources, supervision, data curation, project administration. **Alten Oskay:** resources, supervision, data curation, project administration, writing – review and editing, visualization, validation, conceptualization, investigation, funding acquisition, writing – original draft. **Mert Özen:** methodology. **Atakan Yılmaz:** conceptualization, investigation, funding acquisition, writing – review and editing, visualization, validation, **Mert Özen:** data curation, supervision, resources. **Mert Özen:** conceptualization, investigation, funding acquisition, writing – original draft, writing – review and editing, visualization, validation. **Mert Özen:** project administration, resources, supervision, data curation. **Aylin Köseler:** investigation, conceptualization, supervision, resources, writing – review and editing, writing – original draft, funding acquisition, validation, visualization, project administration, data curation. **İbrahim Türkçüer:** supervision, conceptualization, investigation, funding acquisition, writing – review and editing, visualization, validation. **Mert Özen:** software, formal analysis, project administration, data curation, resources, writing – original draft.

## Ethics Statement

The original study was approved by the Pamukkale University Non‐Invasive Clinical Research Ethics Committee (decision dated June 10, 2021; approval no. 60661). The present Author Reply reports additional analyses of the original individual‐level data set and involved no new participant recruitment or data collection.

## Conflicts of Interest

The authors declare no conflicts of interest.

## Data Availability

The additional analyses reported in this response were performed using the original individual‐level data set underlying the published study. The data are available from the corresponding author upon reasonable request, subject to applicable ethical and institutional requirements.
